# Survival and virulence of *Acinetobacter baumannii* in microbial mixtures

**DOI:** 10.1186/s12866-024-03471-6

**Published:** 2024-09-06

**Authors:** Azam F. Tayabali, Yasmine Dirieh, Emma Groulx, Nusaybah Elfarawi, Sabrina Di Fruscio, Kristina Melanson, Houman Moteshareie, Mustafa Al-Gafari, Martha Navarro, Stéphane Bernatchez, Zerihun Demissie, Valar Anoop

**Affiliations:** 1 Biotechnology Laboratory, Environmental Health Science and Research Bureau, Environmental and Radiation Health Sciences Directorate, Healthy Environments and Consumer Safety Branch, Health Canada, Ottawa, ON K1A 0K9 Canada; 2 Scientific Services Division, Bureau of Chemical Safety, Food Directorate, Health Products and Food Branch, Health Canada, Ottawa, ON K1A 0K9 Canada; 3 Biotechnology Sections 1 and 2, New Substances Assessment and Control Bureau, Safe Environments Directorate, Healthy Environments and Consumer Safety Branch, Health Canada, Ottawa, ON K1A 0K9 Canada

**Keywords:** *Acinetobacter baumannii*, Biotechnology, Cytotoxicity, Immune response, Survival, Virulence

## Abstract

*Acinetobacter* species such as *A. venetianus* and *A. guillouiae* have been studied for various biotechnology applications, including bioremediation of recalcitrant and harmful environmental contaminants, as well as bioengineering of enzymes and diagnostic materials. Bacteria used in biotechnology are often combined with other microorganisms in mixtures to formulate efficacious commercial products. However, if the mixture contained a closely related *Acinetobacter* pathogen such as *A. baumannii* (Ab), it remains unclear whether the survival and virulence of Ab would be masked or augmented. This uncertainty poses a challenge in ensuring the safety of such biotechnology products, since Ab is one of the most significant pathogens for both hospital and community -acquired infections. This research aimed to investigate the growth and virulence of Ab within a mixture of 11 bacterial species formulated as a mock microbial mixture (MM). Growth challenges with environmental stressors (i.e., temperature, pH, sodium, iron, and antibiotics) revealed that Ab could thrive under diverse conditions except in the presence of ciprofloxacin. When cultured alone, Ab exhibited significantly more growth in the presence of almost all the environmental stressors than when it was co-incubated with the MM. During the exposure of A549 lung epithelial cells to the MM, Ab growth was stimulated compared to that in standard mammalian culture media. Cytotoxicity caused by Ab was suppressed in the presence of the MM. Lymphocytes were significantly reduced in mice exposed to Ab with or without MM via intravenous injection. The levels of the splenic cytokines IL-1α, IL-1β, MCP-1, and MIP-1α were significantly reduced 24 h after exposure to Ab + MM. This study demonstrated that the presence of the MM marginally but significantly reduced the growth and virulence of Ab, which has implications for the safety of mixtures of microorganisms for biotechnological applications. Furthermore, these findings expand our understanding of the virulence of Ab during host–pathogen interactions.

**Supplementary Information:**

The online version contains supplementary material available at 10.1186/s12866-024-03471-6.

## Introduction

*Acinetobacter* species are characterized as Gram-negative, capsulated, aerobic cocci. Species such as *A. venetianus*,* A. baylyi*,* and A. guillouiae* have been extensively studied for their applications towards biotechnologies such as bioremediation of wastewater and oil spills, and detoxification of recalcitrant organic and inorganic pollutants [1–3]. The attractiveness of using *Acinetobacter* in biotechnology applications can be partly attributed to its high plasticity and tolerance to harsh conditions. It can thrive under wide temperature and pH ranges, as well as in environments with high salinity, metals or antimicrobial agents [4, 5]. It produces several enzymes such as phenol hydrolases, bio-emulsifiers and biosurfactants, lipases, and wax esters [6]. Contemporary biotechnology products containing *Acinetobacter* species are sometimes formulated as mixtures of microorganisms aimed at increasing their efficacy, stability, and versatility. These microbial mixtures (MM) can include species from diverse genera of prokaryotes or even mixtures of prokaryotes and lower eukaryotes [7, 8].

However *Acinetobacter* species, especially *A. baumannii* (Ab), are also well-recognized nosocomial pathogens, as well as responsible for causing community-acquired infections from natural waters, soil, foods, as well as domestic, wild, and farm animals, and lice [9]. It employs several mechanisms to ensure survival and growth in human hosts including biofilm formation, quorum sensing, metabolic versatility, and genetic plasticity [8, 10, 11]. With its wide range of virulence factors such as outer membrane proteins (OMPs), capsules, lipopolysaccharides, proteases, phospholipases, and metal acquisition systems [12–14], Ab demonstrates antibody resistance to even last-resort antibiotics including colistin and polymyxins [15–19]. As such, Ab is a top international clinical threat and has been included in the World Health Organization’s list of ESKAPE pathogens along with *Enterococcus faecium*,* Staphylococcus aureus*,* Klebsiella pneumoniae*,* Pseudomonas aeruginosa*, and *Enterobacter* species [20].

Several systems in Ab have evolved to sense and adapt to its environment, and establish infections. These systems include two-component regulatory systems (TCS) as signal transducers for sensing environmental or extracellular stimuli [21], quorum sensing systems for monitoring presence of other microorganisms to regulate virulence factor expression [21, 22], outer membrane proteins (e.g., OmpA) with pleiotropic effects including modulating host cellular responses [23], and efflux pumps to remove toxic substances [24].

If Ab were present in microbial mixtures used for biotechnological applications either as contaminants or if it were misidentified as *A. venetianus*,* A. baylyi*, or *A. guillouiae*, it is unknown whether its survival and virulence could be affected by biotechnology application-related constituents and other related microorganisms within the product. The goal of this study was to investigate the growth, survival, and virulence of Ab within a mixture of microorganisms that may be considered for biotechnological applications. To achieve this objective, a microbial mixture (MM) consisting of Risk Group 1 bacteria that could be considered for biotechnological applications was established. The growth and survival of Ab alone or as part of the MM were compared during incubation with various stressors that may represent the environment within a biotechnology product. The virulence and host response of cultured human epithelial cells and mice were also measured following their exposure to Ab alone or as part of the MM. Taken together, our data demonstrated that under the conditions tested, the MM had a statistically significant impact on Ab survival and virulence.

## Materials and methods

### Microorganisms

All bacterial strains were obtained from Cedarlane Laboratories (Burlington, ON, Canada). A mock microbial mixture (MM) was formulated with strains included in Canada’s Domestic Substances List of the Canadian Environmental Protection Act (1999). Although the MM was not developed for any practical application, it consisted of several diverse Risk Group 1 genera, including two *Acinetobacter* species. The microbes in the MM were *A. guillouiae* (American Type Culture Collection (ATCC) 11171), *A. venetianus* (ATCC 31012), *Arthrobacter globiformis* (ATCC 8010), *Bacillus subtilis* (ATCC 6051), *Bacillus licheniformis* (ATCC 12713), *Bacillus amyloliquefaciens* (ATCC 23350), *Pseudomonas fluorescens* (ATCC 13525), *Pseudomonas stutzeri* (ATCC 17588), *Rhodococcus rhodochrous* (ATCC 53968), *Rhodospirillum rubrum* (ATCC 11170), and *Rhodobacter sphaeroides* (ATCC 17023). *Acinetobacter baumannii* (ATCC 9955) was the pathogenic strain used in all studies described here. This non-type strain was isolated from human spinal fluid and is the same strain used in our previous publication [25]. Typical Ab colonies were circular, with a raised elevation, an entire margin, a smooth glistening surface, and were opaque and creamy yellow. Each microorganism was grown separately in tryptic soy broth (TSB; Thermo Fisher Scientific, Mississauga, ON, Canada) and frozen in single-use aliquots at -80 °C in 20% glycerol. An aliquot of each sample was rapidly thawed to determine concentrations by colony enumeration before mixing to formulate the MM.

### Environmental stressors

The test samples consisted of Ab (10^7^ CFU/mL), MM (10^7^ CFU of each strain/mL), or Ab + MM (1:1) and were added to 50 mL culture tubes containing 10 mL of TSB supplemented with stressors as needed. The following stressors were added separately into the different culture tubes containing TSB at 28 °C at pH 7.0: 0.4 mg/L amphotericin B, 0.03 mg/L ciprofloxacin, 20 µM iron chloride, or 10 g/L NaCl. Test temperature conditions were 37–42 °C to reflect core mammalian body and fever temperatures, respectively. Test pH conditions were pH 6.0 or 9.0 adjusted with HCl or NaOH, respectively. These pH’s were selected to correspond to the tissue inflammation microenvironment (pH 5.5-7.0) and those associated with some microbial based biotechnology products for optimal activity of enzymatic additives such as lipases and proteases [26–28]. The controls were grown in TSB alone at 28 °C at pH 7.0. Bacteria were grown with agitation at 350 rpm for 24 h before the cultures were sampled.

### Colony enumeration

Serial dilutions of 10^− 5^, 10^− 6^, 10^− 7^, and 10^− 8^-fold of the original sample were made in TSB (pH 7.0, except for pH tests). One hundred microliters of each dilution was spread on TSB-agar plates in duplicate. The plates were incubated for 24 h at 28 °C (except for temperature tests at 37–42 °C). Colonies of Ab were identified based on colony phenotype, and plates that contained 50 to 300 colonies were enumerated. The accuracy of colony enumeration was validated against enzyme-linked immunosorbent assays (data not shown). However, the results of the immunosorbent assays were inaccurate at Ab concentrations below ~ 10^3^ CFU/mL, which precluded their broad usefulness in this study.

### A549 cell culture, exposure, and viability

Human lung epithelial cells (A549; CCL-185) were obtained from the ATCC and maintained in Dulbecco’s Modified Eagles Medium with 10% fetal bovine serum and penicillin‒streptomycin (100 units/mL and 100 µg/mL, respectively; Dulbecco’s Modified Eagles Medium (DMEM); Thermo Fisher Scientific) in an incubator (95% relative humidity, 5% carbon dioxide, 37 °C) by subculturing with Accutase (Thermo Fisher Scientific, Mississauga, ON, Canada) to maintain 80% confluency. For exposure to bacteria, approximately 10^6^ A549 cells in 1 mL of mammalian culture medium were seeded into each well of a 12-well plate and allowed to adhere overnight. The following day, the culture medium was replaced with fresh medium (control) or fresh medium containing 10^6^ CFU/mL Ab, 10^6^ CFU/mL MM, or Ab + MM (1:1). The plates were incubated at 37 °C for 24 h. Following exposure, 100 µL of the supernatant was plated on TSB agar. Cell viability of A549 was determined using the trypan blue dye exclusion assay according to the manufacturer’s instructions for the Countess™ cell counter (Thermo Fisher). Individual A549 cells were detached from the cell culture with Accutase and mixed with an equal volume of 0.4% trypan blue stain. Following gentle mixing, 10 µL of the stained suspension was loaded onto a Countess™ slide for measurement. The experiments were repeated in triplicate.

### Animal exposures

All procedures involving animals were approved by the Health Canada Animal Care Committee (protocol number HC2022-002). Eight-week-old specific pathogen-free female BALB/c mice were purchased from Charles River Laboratories Inc. (Saint-Constant, Québec) and acclimated for one week. The mice had access to food and sterile water *ad libitum* and were monitored for signs of distress for the duration of the study. Notably, the mice did not exhibit any adverse clinical symptoms. Five mice were used for each treatment group. The mice were injected intravenously through the tail vein with 100 µL of either physiological saline, 10^6^ CFU of Ab, 10^6^ CFU of MM, or Ab + MM (10^6^ CFU each). Animals were weighed at delivery 6 days before exposure (d=-6), on the day of exposure (d = 0), and 1 day after exposure, immediately before necropsy (d = 1).

One day (24 h) after exposure, the mice were placed in a chamber and anaesthetized with 5% (v/v) isoflurane with oxygen at a flow rate of 0.8–1.0 L/min. When animals were unconscious, respiration was regular and shallow, and reflexes were notably absent, blood (approximately 500–1000 µL) was immediately collected by cardiac puncture and transferred to blood collection tubes containing ethylenediaminetetraacetic acid (EDTA). Following blood collection, animals were cervically dislocated prior to tissue collection described below. For leukocyte differentials, blood was diluted 1:1 with Beckmann-Coulter diluent and loaded into a Sysmex XT-2000i V Hematology Analyser.

Splenocytes were isolated from fresh spleens. Following excision, 1/3 of the spleen was diced with a sterile scalpel blade. The tissue pieces were transferred onto a 70 μm nylon filter in a 50 mL tube and crushed with a sterile 3.0 mL syringe plunger while adding ice-cold RPMI-1640 medium + 10% FBS. The cell suspension was centrifuged for 5 min at 450 × *g* at 4 °C, and red blood cells (RBCs) were lysed with red blood cell (RBC) lysis buffer (eBiosciences, San Diego, CA). The resulting cell suspension was counted with a hemocytometer and adjusted to 1.5 × 10^7^ cells/mL. The cells were stained with Horizon™ Fixable Viability Stain (FVS 510; BD Biosciences, San Jose, CA) and incubated with Fc Block (BD Biosciences) and a mixture of fluorochrome-conjugated antibodies (CD3 Pacific Blue, CD19 AlexaFluor 647, and Ly6G FITC). The data were acquired on a BD LSRFortessa™ flow cytometer using BD FACSDiva™ software (BD Biosciences). Cell populations were identified using a sequential gating strategy on live singlet cells.

Another 1/3 of the spleen was used for measuring cytokines using a multianalyte profiling system (Bio-Plex 200; Bio-Rad, Hercules, CA) as described previously [29]. The levels of twenty-three cytokines/chemokines (Eotaxin, G-CSF, GM-CSF, IFN-γ, IFN-α, IFN-β, IL-2, IL-3, IL-4, IL-5, IL-6, IL-9, IL-10, IL-12 (p40), IL-12 (p70), IL-13, IL-17 A, KC, MCP-1 (MCAF), MIP-1α, MIP-1β, RANTES, and TNF-α) in the spleen were measured according to the manufacturer’s instructions. Briefly, spleen samples were homogenized in lysis buffer (Bio-Plex Cell Lysis Kit, Bio-Rad) using a hand-held microtube homogenizer on ice and then centrifuged at 4500 × g for 4 min, after which the supernatant was collected. Magnetic beads coupled to specific cytokine/chemokine antibodies were pipetted into the wells of a 96-well plate and washed with Bioplex wash buffer. Cytokine standards (Bio-Rad) and spleen homogenates were added to the wells and incubated for 30 min with gentle shaking. The wells were washed 3 times with wash buffer before 25 µL/well of detection antibody was added, and the plates were incubated for 30 min. After another cycle of washing, 50 µL/well of streptavidin-conjugated phycoerythrin was added, and the plate was shaken for 10 min. The wells were washed again, and the beads were resuspended in 125 µL/well of assay buffer. After 1 min of gentle shaking, the beads were counted and analysed with a Bio-Plex 200 Array System (Bio-Rad).

To measure the clearance of Ab from tissues, 1 cm^3^ pieces of liver, kidney, and the remaining 1/3 of the spleen were each homogenized with 1 mL of sterile saline with a hand-held microtube homogenizer. Serial dilutions of each tissue sample were plated onto TSB-agar plates, and the CFU content was determined after incubation at 37 °C for 24 h.

### Statistical analyses

The results were tabulated using Microsoft Excel and compared by one or two-way analysis of variance (ANOVA) followed by an appropriate post hoc analysis by correcting for multiple comparisons using either statistical hypothesis testing (Tukey’s or Holm‒Sidak) or by controlling the false discovery rate (two-stage step-up method of Benjamini, Krieger and Yekutieli) using GraphPad Prism version 7.0 software. All the data are expressed as the means ± standard deviations. For spleen cytokines, each cytokine was analysed individually, without assuming a consistent standard deviation. A value of *p* < 0.05 was considered to indicate statistical significance.

## Results and discussion

There is a paucity of information on the growth, persistence, and virulence of Ab within mixed microbial communities. While previous research has expanded our understanding of Ab quorum sensing when co-incubated with specific pathogens such as *Staphylococcus aureus*, *Pseudomonas aeruginosa*, and *Klebsiella pneumoniae* [30, 31], we aimed to investigate how the growth and virulence of Ab may be influenced when it is present within a complex MM mimicking those used for biotechnological applications.

### Growth and environmental stress

**Fig. 1:**
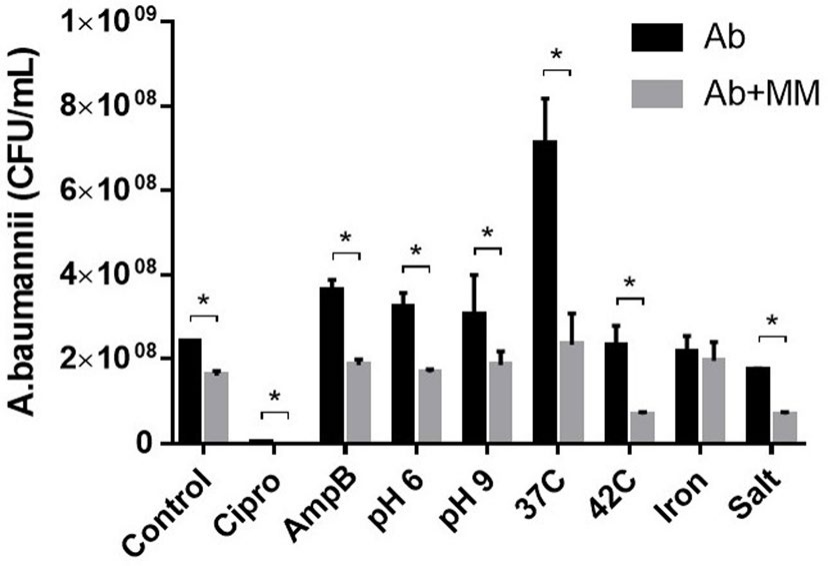
Growth of Ab in response to environmental stressors. Ab was cultured with and without MM under various environmental conditions: Cipro: ciprofloxacin, AmpB: amphotericin B, pH 6 or 9, elevated temperatures, iron chloride, and sodium chloride. The control conditions were TSB at 28 °C and pH 7.0. Colonies that corresponded to Ab were enumerated after a 24 h incubation with an agitation of 350 rpm. Error bars indicate the standard deviation of four measurements from two separate experiments. Asterisks indicate significant differences calculated using 2-way ANOVA with environmental stressors and the presence of MM as variables, followed by controlling for the false discovery rate using the two-stage step-up method of Benjamini, Krieger and Yekutieli (*p* < 0.05)

The growth of Ab in the MM was assessed under various environmental stress conditions. Figure 1 shows a small but statistically significant difference (2 × 10^8^ and 1.6 × 10^8^ CFU/mL, respectively) between Ab grown alone and within the MM under control conditions (28 °C, pH 7.0 in TSB).

The growth of Ab was significantly diminished when present within the MM under seven of the eight environmental conditions tested, but no significant effect was observed when iron chloride was added to the culture medium (Fig. 1). Iron is required for pathogenic virulence and environmental survival. Ab produces several siderophores, such as acinetobactin, baumannoferrin, and fimsbactin [14, 32]. Acinetobactin in particular is highly conserved in clinical isolates, and Acinetobactin knockout mutants show markedly reduced virulence compared to mutants of baumannoferrin and fimsbactin. In the experiments presented here, acinetobactin may have contributed to the observed growth advantage of Ab compared to the microbes present in the MM. Similar suppressive effects have been observed towards commensal skin bacteria *Staphylococcus hominis* and *Corynebacterium striatum* [33].

Among the conditions tested, the inclusion of ciprofloxacin had the greatest inhibitory effect (> 99%) on Ab grown alone or within the MM. Ciprofloxacin, a secondary fluoroquinolone, inhibits topoisomerase type II (i.e., DNA gyrase) and topoisomerase IV, preventing DNA unfolding during cell division. These results corroborate our previous study demonstrating that ciprofloxacin inhibited Ab growth at a concentration of 0.38 µg/mL and that Ab strain 9955 does not harbor mutations in the quinolone resistance-determining regions of *parC* and *gyrA* [25]. The minimum inhibitory concentration of ciprofloxacin for Ab according to the European Committee on Antimicrobial Susceptibility Testing (EUCAST) is 0.001 µg/mL, which is well below our test concentration of 0.03 µg/mL.

The most important growth difference was observed when the cultures were incubated at 37 °C, which resulted in a 3-fold difference between Ab grown alone and Ab grown within the MM. Although the difference was quantitatively marginal, it was statistically significant (7 × 10^8^ for Ab and 2.3 × 10^8^ for Ab + MM; *p* < 0.0001). Recognized for its strong quorum sensing capacity [34] compared to other non-pathogenic *Acinetobacter* species such as *A. guillouiae* and *A. venetianus* included in the MM, Ab has adapted for growth in human hosts and grows optimally at 37 °C [25] while the other species grow optimally at lower temperatures (data not shown). As a human pathogen, Ab has adapted to growth with human hosts and optimally thrives at 37 °C and pH 7 [25]. If a human infection resulted from exposure to an MM containing an Ab, it is probable that the Ab would outcompete the other microorganisms. Collectively, these findings demonstrate the adaptability of Ab under various environmental conditions, which is consistent with its known plasticity for survival [11, 35].

### Cytotoxicity toward A549 epithelial cells

**Fig. 2:**
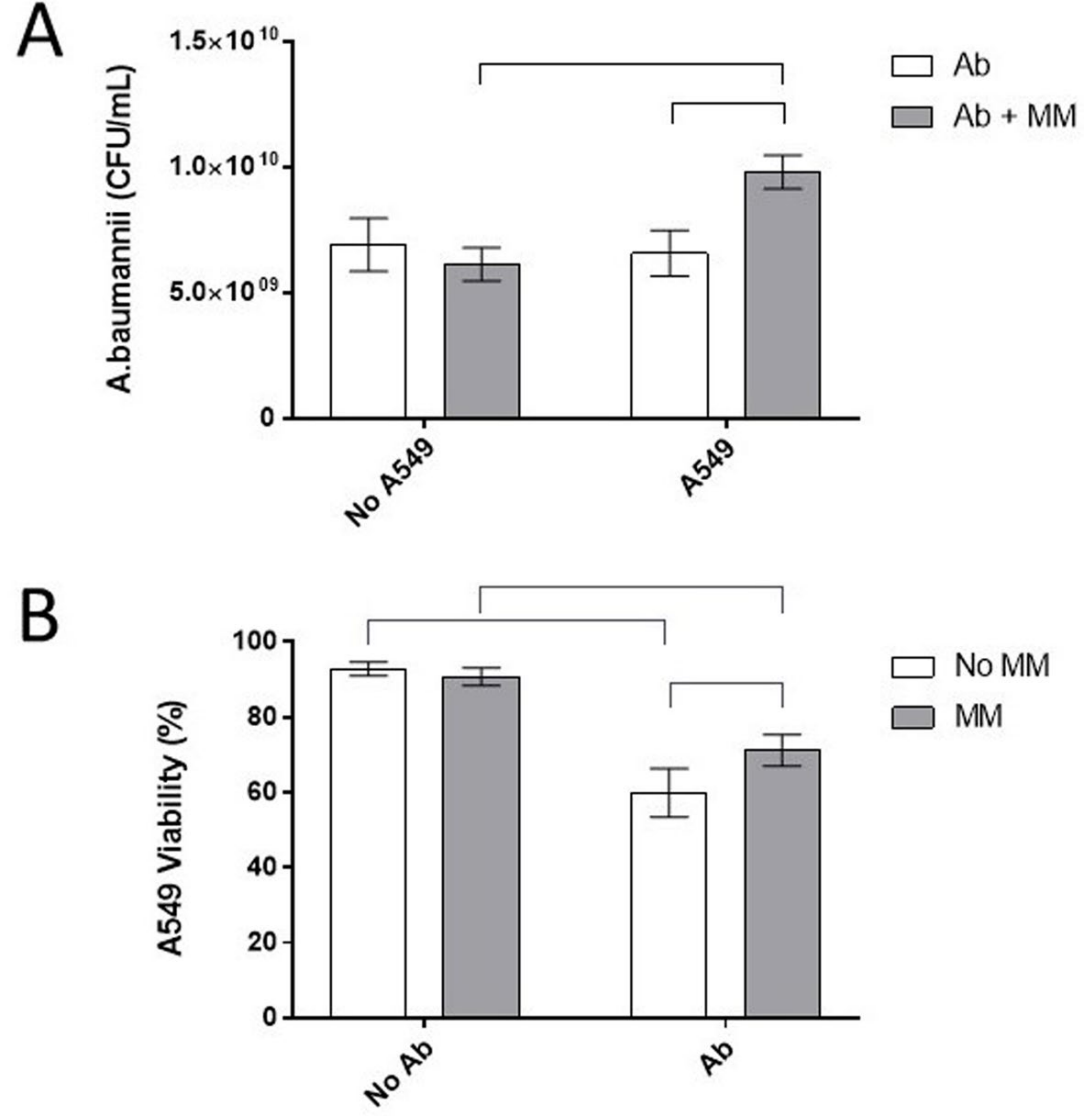
Growth and cytotoxicity of Ab during A549 cell exposure: A549 lung epithelial cells were exposed to Ab with or without MM for 24 h at 37 °C. The bacterial growth of the exposed cells was assessed using colony enumeration (**A**), and the viability of the A549 cells was assessed using a trypan blue dye exclusion assay (**B**). Error bars indicate the standard deviation of six measurements from three separate experiments. Statistical differences are indicated with horizontal bars and were calculated using 2-way ANOVA with the presence of A549 cells and MM as variables, followed by Tukey’s post hoc analysis (*p* < 0.05)

The influence of Ab coculture with A549 lung epithelial cells was investigated with Ab alone, within the MM, and with the MM alone. Figure 2A presents the colony-forming unit (CFU) counts of Ab with and without A549 cells. There was no significant difference in the growth of Ab alone or Ab within the MM in DMEM + 10% FBS, contrary to what had been observed in TBS at 37 °C (Fig. 2A (No A549) and Fig. 1). In contrast, the growth of Ab alone and within the MM improved upon exposure of A549 cells (Fig. 2A (A549)). Although these changes were minor, it is plausible that Ab modulated its growth under complex conditions that mimic infection. A549 cell viability was reduced by 30–40% when the cells were exposed to Ab alone or within the MM (Fig. 2B). There was a significant albeit small (10%) difference in the viability of A549 cells following exposure to Ab alone or within the MM, which is consistent with the increased growth observed in Fig. 2A. Ab expresses several virulence factors, including phospholipases and hemolysins, in their outer membrane vesicles [36], as well as determinants involved in intracellular pathogenesis [37]. Furthermore, gene expression changes during infection or in a simulated environment have revealed alterations in iron transport, nutritional changes, capsule modifications, motility, efflux pumps, and osmotic stress adaptations [38–40]. We did not investigate the altered expression of any of these virulence determinants in the presence of the MM, although this could be an informative topic of future research.

### Murine sepsis model

Mice were intravenously exposed to Ab to simulate the worst-case scenario of septicemia resulting from organ damage. A key difference between intravenous tail vein exposure and septicaemia is that the latter typically arises as a gradual poisoning of the blood and not as a bolus administration [41]. Nevertheless, we expected that exposure via the tail vein would circulate the bacteria to multiple organs and clarify the influence of the MM on Ab virulence.

There were no significant changes in the whole body weight of the mice over the 24-h exposure period (Figure S1). Following necropsies, the kidney, liver, and spleen were homogenized, and each was spread onto TSB media. Some animals displayed an increased number of colonies within their kidney and liver tissues, but there was no significant change in the overall CFU count for any treatment (Figure S2).

In general, the number of colonies from the spleen (Figure S2C) was reduced by at least tenfold regardless of the treatment compared to that from the kidney (Figure S2A) and liver (Figure S2B), except for one animal treated with Ab + MM, which yielded 25 colonies (Figure S2). Nevertheless, only treatments containing Ab alone or within the MM resulted in splenic colonies. Exposure to the MM did not lead to the accumulation of colonies in the spleen (Figure S2). These data suggested that the microbes present in the MM can be efficiently inactivated and/or cleared. The viable Ab recovered from the spleen may represent bacteria taken up by intracellular innate immune cells such as marginal zone phagocytes, which is typical during early bacterial infections [42]. This observation is consistent with a study that showed rapid Ab dissemination and replication within multiple organs, including the spleen, within 24 h after entry into the bloodstream [43].

### Hematological analysis

**Fig. 3:**
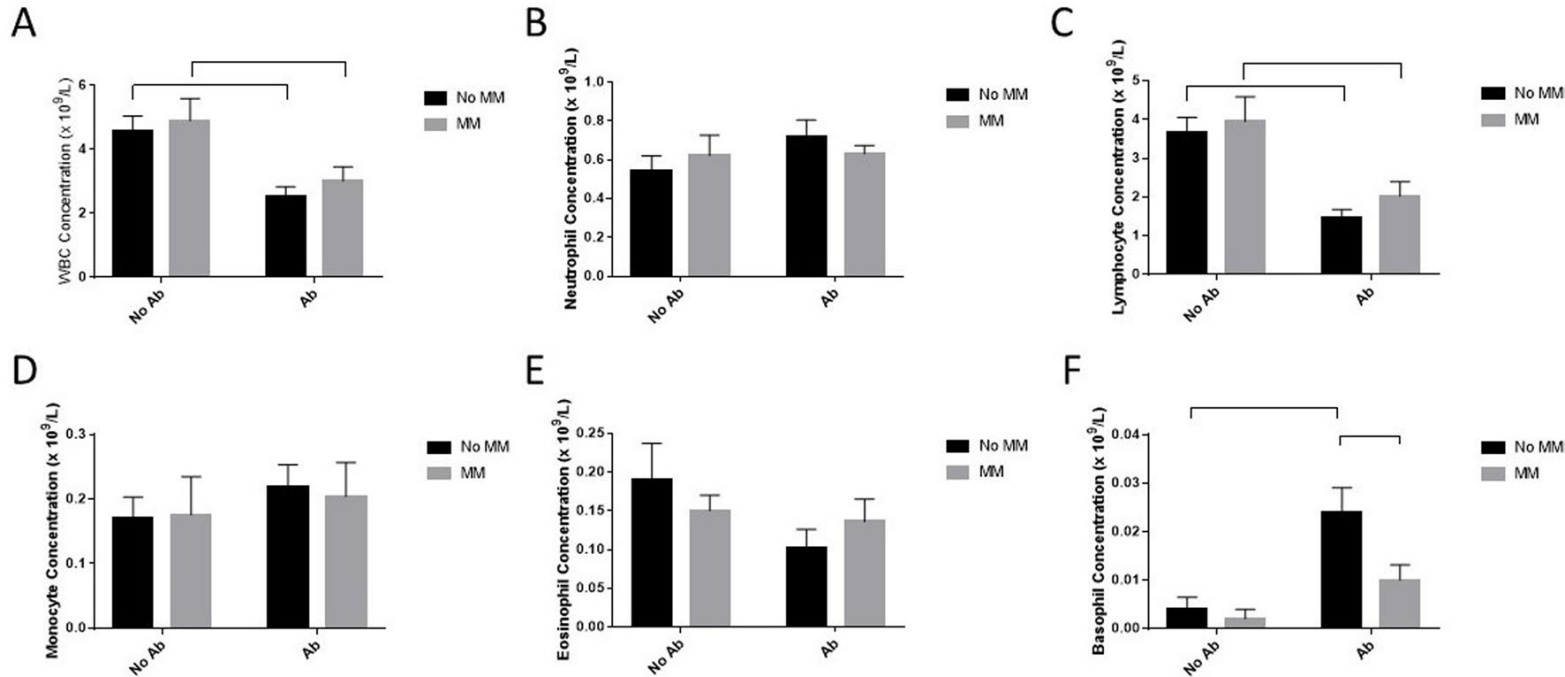
Peripheral blood leukocyte changes in mice: Following exposure, the mice were terminally bled by cardiac puncture. Blood was analysed for leukocyte differences using a haematology analyser. Error bars indicate the standard deviation of five replicate mice per treatment. Statistical differences are indicated with horizontal bars and were calculated using a 2-way ANOVA with the presence of Ab and the presence of MM as variables, followed by Tukey’s post hoc analysis (*p* < 0.05)

Peripheral blood was examined using a hematological analyser to detect alterations in leukocyte levels. As shown in Fig. 3, the number of leukocytes was unchanged following exposure to the MM alone compared to basal levels. No significant changes were observed in the levels of neutrophils, monocytes, or eosinophils (Fig. 3B, D, E). Total white blood cells (WBCs) were reduced by 44% following exposure to Ab alone and by 39% following exposure to Ab within the MM (Fig. 3A). Lymphocyte numbers were also reduced following exposure to Ab alone (41%, *p* = 0.0133) and within the MM (51%, *p* = 0.0299; Fig. 3C). Interestingly, basophil levels were elevated sixfold (*p* = 0.0036) only following exposure to Ab alone (Fig. 3F). In a study by Xie and colleagues, basophil levels measured by hemocytometry increased tenfold 8 h following intraperitoneal exposure to [44]. A review by Chen summarized the innate immune response to Ab infection [43]. Early innate immunity is characterized by elevated levels of neutrophils and macrophages, yet Ab employs mechanisms to evade these cells. Elevated basophils could represent a secondary immune mechanism. An alternative explanation could be that elevated peripheral basophils represent an instrumentation artefact known as pseudobasophilia. Hematology analysers typically measure basophils based on their resistance to acidolysis. Cells isolated from a pathological sample may be misclassified as basophils if a population of cells exhibits heightened resistance to acid lysis [45]. Depending on the instrument and on the specific pathology, both monocytes and dysgranulopoietic neutrophils have previously been enumerated as basophils [46]. Manuel and colleagues suggested that pseudobasiphilia is caused by atypical lymphocytes, which are characterized by microscopy as having almost no cytoplasm [47]. To further investigate this possibility, blood samples were smeared on glass slides and manually enumerated by microscopy after Wright’s staining. The slides did not reveal any cells resembling basophils in any treatment and instead showed cells with very low cytoplasm (Figure S3) resembling those observed by Manuel and colleagues. This observation confirmed that the haematology analyser misclassified these samples. Nevertheless, it is intriguing that exposure to Ab mixed with the MM did not result in pseudobasophilia in the mice to the same extent as exposure to Ab alone.

### Cytokine/chemokine analysis

**Fig. 4:**
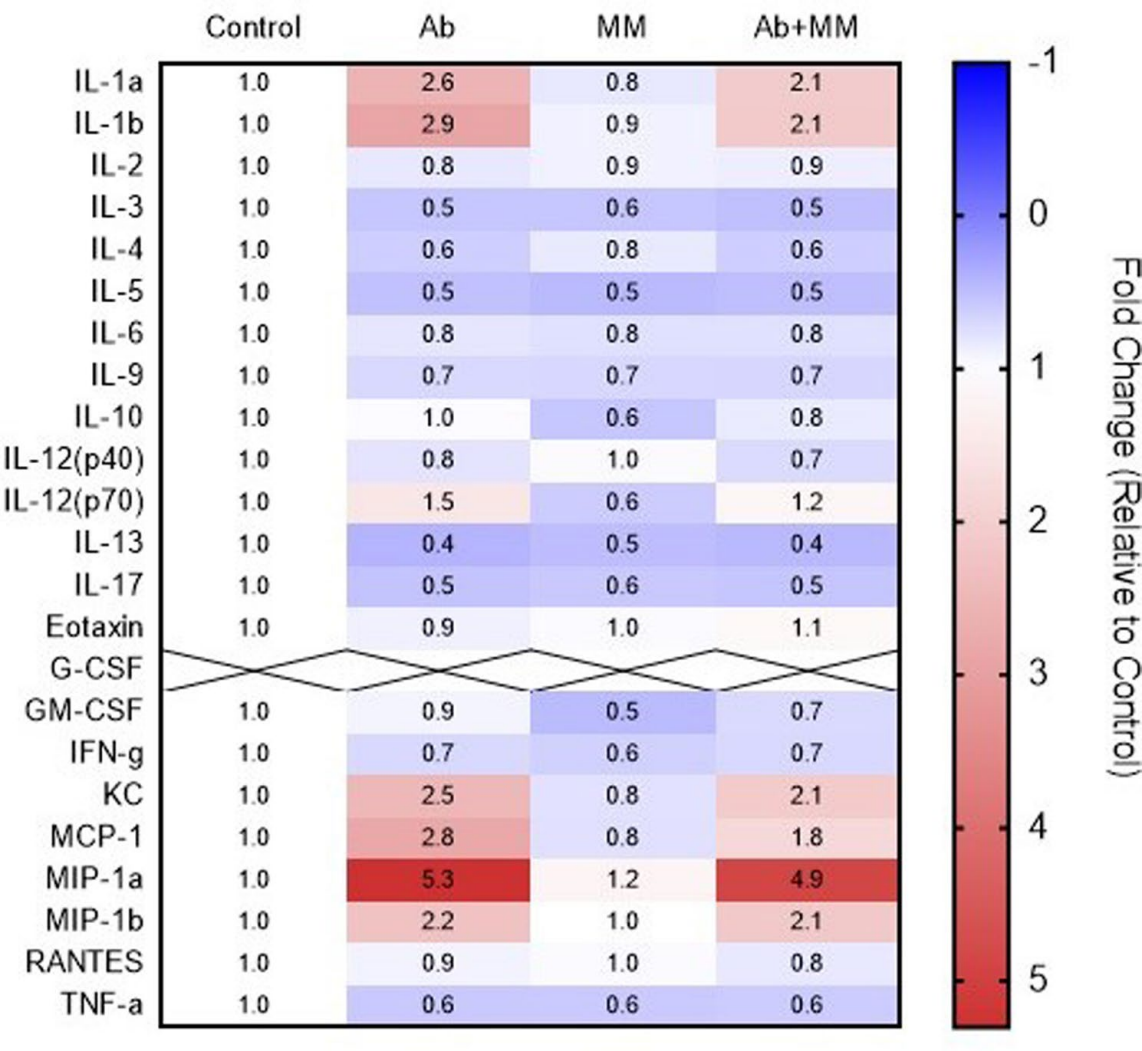
Spleen cytokine changes in mice: Following exposure, the mice were terminated by exsanguination. The spleen was excised, and a portion was homogenized and subjected to cytokine/chemokine measurements using the xMAP method. The heatmap indicates the fold change of individual cytokines with respect to spleens from control (vehicle-treated) mice. Each value represents the mean from 5 replicate mice

To measure cytokine/chemokine levels in the spleen, a 23-plex xMAP assay was conducted. The data normalized to control concentrations are summarized on a heatmap (Fig. 4). All markers yielded data except for G-CSF, which was not detected at sufficiently high levels (indicated with X’s in Fig. 4). Exposure to Ab resulted in altered expression of several cytokines compared to the control levels (Fig. 4; Table 1). Among these cytokines, significant differences in the expression of IL-1α, IL-1β, MIP-1α, and MCP-1 were noted following exposure to Ab and Ab within the MM (Table 2). These differences were relatively minor, as demonstrated by the low t ratios and the heatmap (Table 2; Fig. 4). Notably, no significant changes were observed in the expression of cytokines/chemokines following exposure to the MM or to the control (i.e., physiological saline). These results indicate that the induction of inflammation is consistent with a 24-hour innate immune response, as observed in previous studies in peripheral mouse blood [43, 44, 48]. The data also suggested that the MM had a marginal attenuating effect on proinflammatory signal expression. Alternatively, the attenuated response could be due to temporal changes in the expression of these signals since this study investigated only a single timepoint, 24 h postexposure.

**Table 1:** Differential cytokine/chemokine protein expression between control and ab treatments

	*P* value	Ab Mean	Control Mean	Difference	SE of difference	t ratio	df	Adjusted *P* Value
*IL-1a*	< 0.0001	2.557	1	1.557	0.1202	12.95	8	< 0.0001
*IL-1b*	< 0.0001	2.88	1	1.88	0.09661	19.46	8	< 0.0001
*IL-12(p40)*	0.0016	0.7864	1	-0.2136	0.0459	4.653	8	0.0259
*KC*	< 0.0001	2.479	1	1.479	0.1727	8.568	8	0.0005
*MCP-1*	< 0.0001	2.785	1	1.785	0.1315	13.57	8	< 0.0001
*MIP-1b*	< 0.0001	2.241	1	1.241	0.06405	19.38	8	< 0.0001
*MIP-1a*	< 0.0001	5.286	1	4.286	0.09281	46.18	8	< 0.0001

**Table 2:** Differential cytokine/chemokine protein expression between ab and ab + MM treatments

	*P* value	Ab + MM Mean	Ab Mean	Difference	SE of difference	t ratio	df	Adjusted *P* Value
*IL-1a*	0.0009	2.058	2.557	-0.499	0.09756	5.115	8	0.0172
*IL-1b*	< 0.0001	2.094	2.88	-0.7858	0.09959	7.89	8	0.0011
*MCP-1*	0.0001	1.802	2.785	-0.9828	0.1408	6.983	8	0.0024
*MIP-1a*	0.0003	4.864	5.286	-0.422	0.07022	6.009	8	0.0064

### Splenic leukocyte levels

**Fig. 5:**
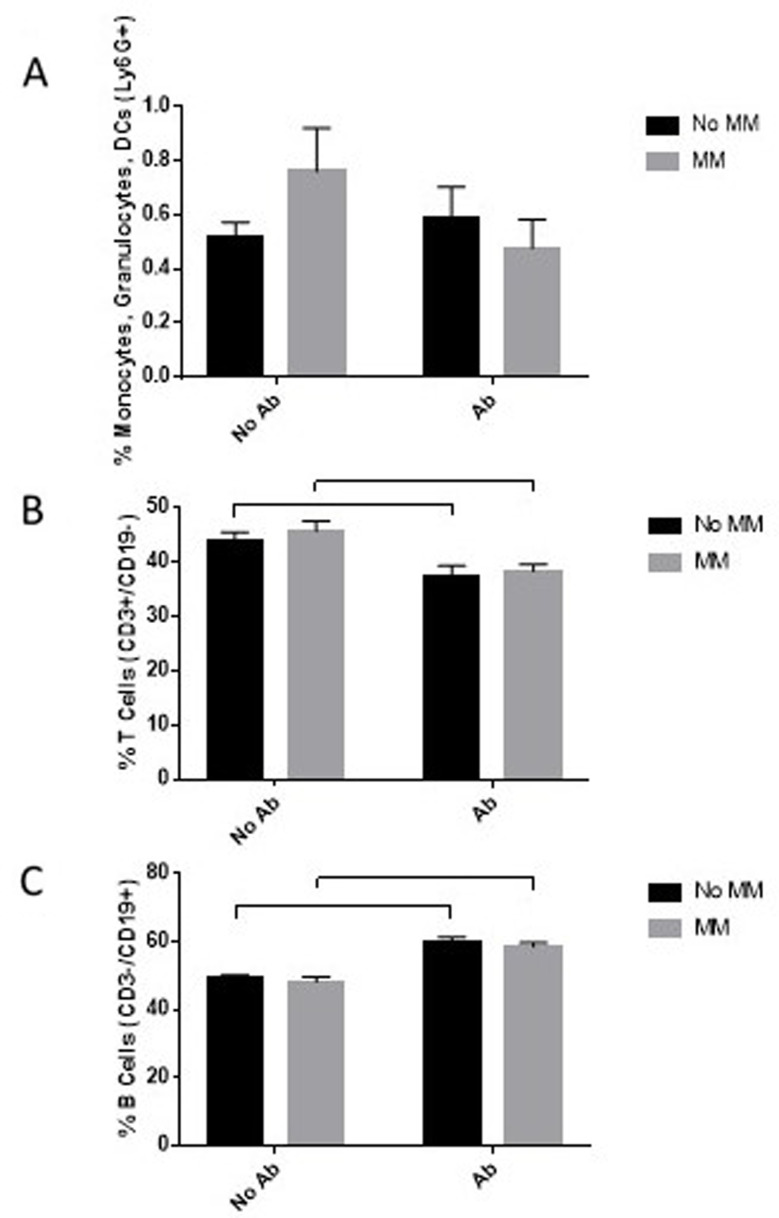
Splenic leukocyte changes in mice: Following exposure, the mice were terminated by exsanguination. The spleen was excised, and a portion was used to prepare a single-cell suspension. Live leukocytes were measured with specific antibodies and flow cytometry. Error bars indicate the standard deviation from the spleens of five replicate mice per treatment. Statistical differences are indicated with horizontal bars and were calculated using a 2-way ANOVA with the presence of Ab and the presence of MM as variables, followed by Tukey’s post hoc analysis (*p* < 0.05)

Analysis of viable spleen leukocytes was conducted by flow cytometry to determine alterations in immune cell populations following exposure. No changes were observed in the number of Ly6G + cells (Fig. 5A). In the spleen, these cells are predominantly neutrophils but also inflammatory monocytes, macrophages, and myeloid-derived suppressor cells. However, after exposure to either Ab or Ab within the MM, the percentage of splenic CD3 + T cells was reduced by approximately 5% (Fig. 5B), while the percentage of CD19 + B cells was increased by approximately 10% (Fig. 5C). These lymphocytes are involved in the adaptive immune response, although adaptive immunity typically develops well after 24 h postinfection. Nevertheless, no significant differences were observed between any of the exposures to Ab or to Ab within the MM.

### Conclusions and implications

Under the conditions investigated, our data indicate that the persistence and virulence of the Ab strain ATCC 9955 were attenuated in the presence of other bacteria. Growth of Ab with environmental stressors was notably better in the absence of MM. Cytotoxicity of Ab towards A549 cells was suppressed in the presence of MM. In mouse exposures, the presence of MM caused lower lymphocyte and inflammatory cytokine levels compared to Ab exposures alone. Collectively, these results have implications for testing MMs used in biotechnological applications. If Ab were present in a biotechnology MM, its effects may be underestimated if testing MMs. This study focused on a single Ab isolate, so future studies could investigate additional clinical and environmental isolates and study the expression of key virulence genes such as those involved in two-component regulatory systems, quorum sensing, and biofilm formation. A similar approach could also be applied to other pathogens that are closely related to those used in biotechnology.

## Electronic supplementary material

Below is the link to the electronic supplementary material.

Supplementary Material 1

Supplementary Material 2

Supplementary Material 3

Supplementary Material 4

## Data Availability

The datasets used and/or analysed during the current study are available from the corresponding author upon reasonable request.
