# Supplementary figures and images for: Survival and virulence of Acinetobacter baumannii in microbial mixtures
Source: BMC Microbiol. 2024 Sep 6;24:324. doi: 10.1186/s12866-024-03471-6 (PMC11378493; doi:10.1186/s12866-024-03471-6)

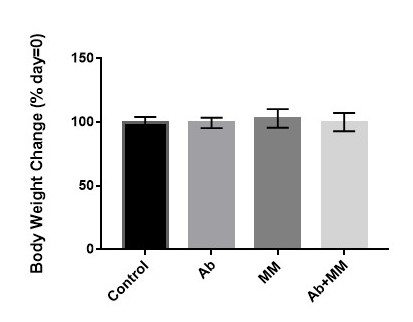

Supplement: Supplementary file 1 — Supplementary Material 1 [file 12866_2024_3471_MOESM1_ESM.jpg]

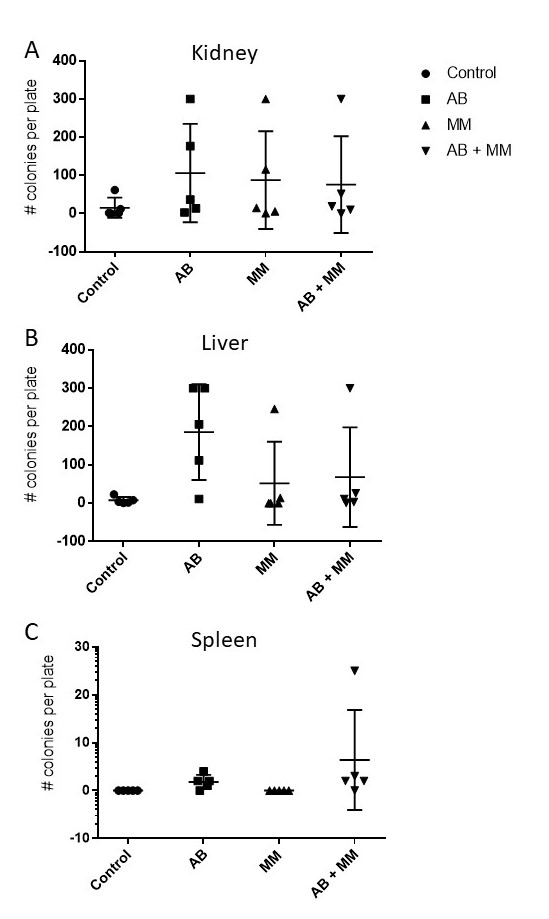

Supplement: Supplementary file 2 — Supplementary Material 2 [file 12866_2024_3471_MOESM2_ESM.jpg]

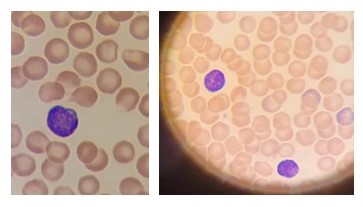

Supplement: Supplementary file 3 — Supplementary Material 3 [file 12866_2024_3471_MOESM3_ESM.jpg]
