# Supplementary material for: Survival and virulence of Acinetobacter baumannii in microbial mixtures
Source: BMC Microbiol. 2024 Sep 6;24:324. doi: 10.1186/s12866-024-03471-6 (PMC11378493; doi:10.1186/s12866-024-03471-6)
Supplement: Supplementary file 4 — Supplementary Material 4 [file 12866_2024_3471_MOESM4_ESM.docx]

**Figure S1: Body weight changes in mice:** Mice were weighed before (day=0) and after exposure (day=1). The graph shows the average change in body weight as a percentage of weight before treatment. Error bars indicate the standard deviation from the spleens of five replicate mice per treatment. No significant differences were detected using 1-way ANOVA (p<0.05).

**Figure S2: Bacterial colonies from mouse tissues:** Following exposure, the mice were terminated by exsanguination. Various tissues were excised, and a portion of each sample was homogenized and spread onto agar plates for colony enumeration. Error bars indicate the standard deviation of colonies from the tissues of five replicate mice per treatment. No significant differences were detected using 1-way ANOVA (p<0.05).

**Figure S3: Atypical lymphocytes from Ab-treated mice:** Following exposure, the mice were terminally bled by cardiac puncture. Blood was smeared onto glass microscopy slides, fixed with 10% (v/v) formalin, and stained with a modified Wright stain (Epredia™ Richard-Allan Scientific™). Bright-field microscopy revealed atypical lymphocytes, which were not detected in blood from mice treated with either vehicle (physiological saline) or MM alone.
